# Bilateral papillitis and unilateral focal chorioretinitis as the presenting features of syphilis

**DOI:** 10.1186/s12348-015-0045-0

**Published:** 2015-06-05

**Authors:** Christy Elizabeth Benson, Mohamed Kamel Soliman, Alexander Knezevic, Daisy Ding Xu, Quan Dong Nguyen, Diana V Do

**Affiliations:** Stanley M Truhlsen Eye Institute, University of Nebraska Medical Center, 3902 Leavenworth Street, Omaha, NE 68105 USA; Department of Ophthalmology, Assiut University Hospital, Al Gamaa St, Assiut, 71516 Egypt; Tenth People’s Hospital, 301 Yanchang Road, Shanghai District, Shanghai, 200072 China

**Keywords:** Syphilis, Retinitis, Chorioretinitis, Uveitis, Panuveitis, Papillitis

## Abstract

**Background:**

Syphilis is a multisystem bacterial infection caused by *Treponema pallidum*. The incidence of infection in the United States has risen by more than 75% since the year 2000, when it was at a low of 2.1 per 100,000 people. Ocular involvement may occur in any stage of infection and may present in a variety of ways, with posterior uveitis being the most common manifestation. We report a case of ocular syphilis infection with an unusual presentation of bilateral non-granulomatous panuveitis with papillitis and unilateral focal chorioretinitis.

**Findings:**

This is a retrospective case report with literature review. A 39-year-old Caucasian female presented with a 2-week history of bilateral ocular flashes and left eye pain. Dilated fundus examination revealed mild optic disc edema in both eyes, the right eye more than the left. In the left eye, there was an area of retinal elevation and whitening involving the peripheral retina. Fluorescein angiography, B-scan ultrasonography, and ocular coherence tomography were performed, and laboratory tests were ordered based on the clinical presentation. After rapid plasma reagin (RPR) and fluorescent treponemal antibody absorption (FTA-Abs) were positive, syphilitic uveitis was confirmed, and the patient was admitted for a 14-day course of high-dose intravenous penicillin G.

**Conclusions:**

The first signs and symptoms of syphilis may be ocular, which can lead to a diagnostic challenge. A high index of suspicion is the key for early diagnosis of ocular syphilis. Prompt treatment with intravenous penicillin G is highly effective in resolving the infection.

## Findings

Syphilis is a multisystem bacterial infection caused by the spirochete *Treponema pallidum* [1]. It is primarily a sexually transmitted disease; however, contacts with an infected lesion and blood transmission are also potential routes of infection. The classic clinical course of acquired syphilis is divided into four stages: primary, secondary, latent, and tertiary syphilis [2]. The eye can be affected in any stage of infection and virtually all ocular tissues can be affected.

Uveitis occurs in approximately 10% of cases of secondary syphilis and in up to 5% of cases who have progressed to tertiary syphilis [3,4]. The uveitis that occurs with syphilis may be granulomatous or non-granulomatous [5], and it can affect one or both eyes in the anterior, intermediate, or posterior segments.

Syphilis earns its name as the ‘great masquerader’ in its ability to produce a myriad of signs and symptoms that may mimic various diseases [6]; therefore, syphilis should be kept in the differential diagnosis of ocular inflammation. Unfortunately, when ophthalmologic involvement becomes the presenting signs and symptoms, the proper diagnosis and treatment may be delayed [3,7]. Such a delay in treatment may result in irreversible visual loss and significant systemic morbidity.

### Case report

A 39-year-old healthy Caucasian female from rural Nebraska presented with a 2-week history of bilateral flashes and left eye pain. This was associated with redness in both eyes. The patient was generally healthy with no history of eye discharge or trauma. An extensive review of symptoms was performed, which was negative. There was no previous history of eye diseases or surgery. The patient had no chronic medical conditions.

**Figure 1:**
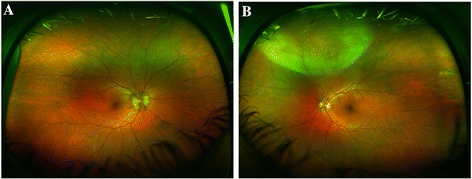
**Fundus photograph of the right and left eyes. (A)** Wide field color fundus photograph of the right eye: blurred optic disc margin. **(B)** Wide field color fundus photograph of the left eye: well-defined area of whitening involving the peripheral superonasal quadrant with slight haziness extending from that area up to the upper margin of disc and upper temporal arcade. The disc margin is ill-defined.

On examination, the patient’s visual acuity was 20/20 in both eyes. Slit-lamp examination revealed 1+ conjunctival injection in the right eye and 2+ in the left eye. The corneas were clear in both eyes. Anterior chamber examination using slit-lamp biomicroscopy showed 0.5+ and 2+ cells (non-granulomatous) and flare in the right and left eyes, respectively. The pupils were equally round and reactive to light without evidence of relative afferent papillary defect in either eye. There was trace nuclear sclerosis and pigment deposits in the crystalline lens of the left eye and 1+ vitreous cell in the left eye. Intraocular pressure was within normal limits in both eyes. Fundus examination revealed mild optic disc edema in both eyes, the right eye more than the left, with 0.3 cup-to-disc ratio of both eyes. There was a subtle retinal elevation and well-defined area of whitening involving the retina in the left eye. The macula and vessels showed no visible abnormalities in either eye (Figure 1A,B).

**Figure 2:**
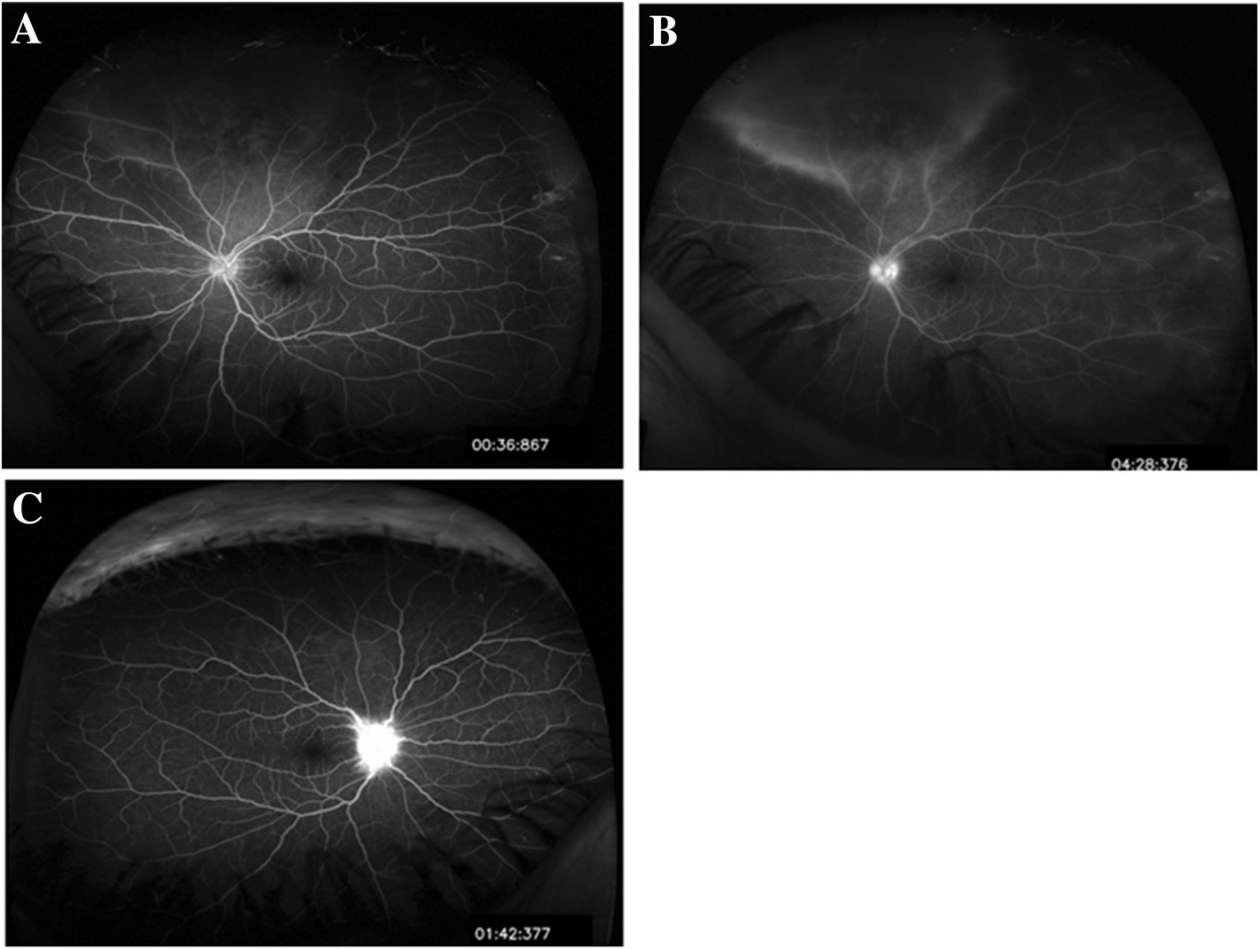
**Fluorescein angiography of the left and right eyes. (A)** Fluorescein angiography of the left eye: early hyperfluorescence corresponding to the area of retinal whitening and haziness. **(B)** Fluorescein angiography of the left eye: late frame shows perivascular leakage in the same area (vasculitis) together with late hyperfluorescence of the disc. **(C)** Fluorescein angiography of the right eye: late hyperfluorescence and leakage from the disc.

**Figure 3:**
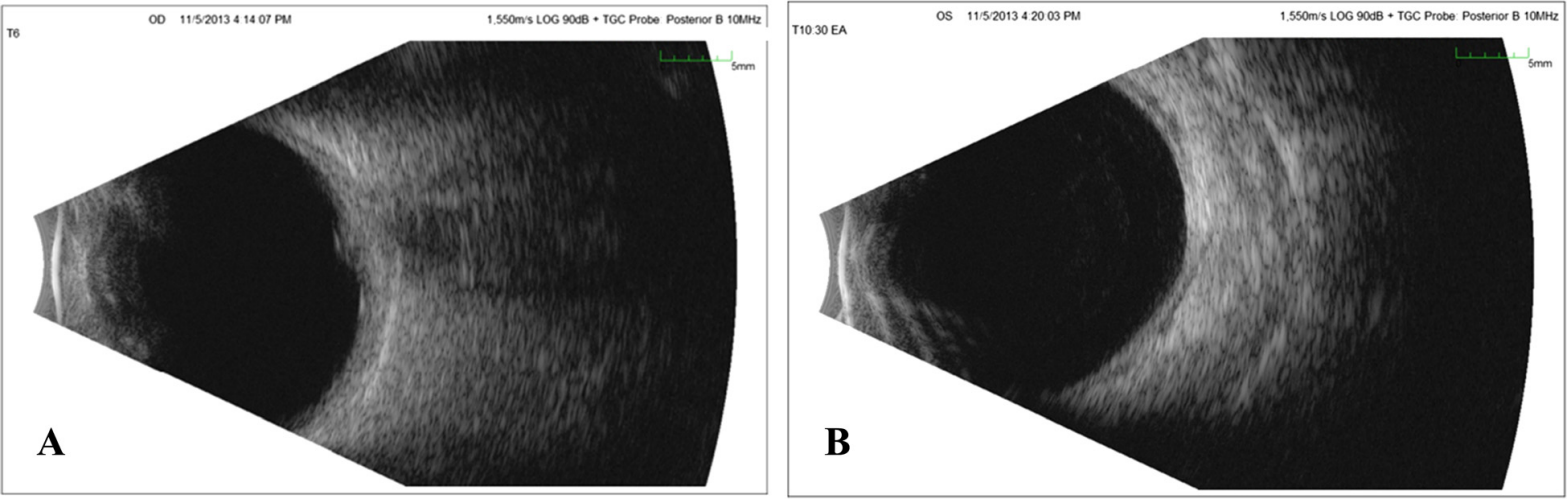
**B-scan ultrasound of the right and left eyes. (A)** B-scan ultrasound of the right eye: elevation of optic nerve head. **(B)** B-scan ultrasound of the left eye: no visible elevation could be appreciated in the superonasal quadrant of the left eye.

**Figure 4:**
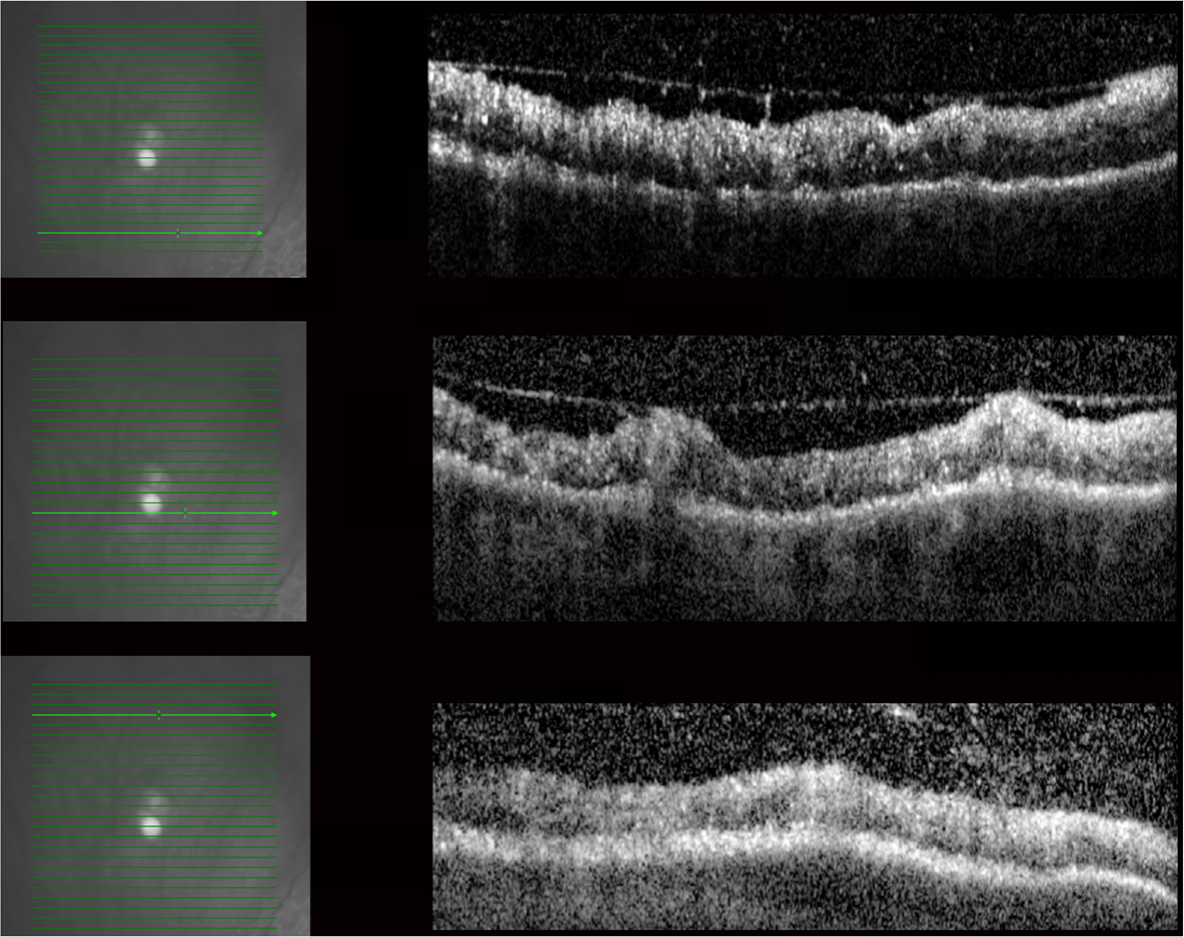
**OCT of the lesion: irregular retinal contour with areas of retinal elevation.** The individual retinal layers could not be distinguished due to infiltration with multiple hyperreflective dots. Diffuse thickening at the retinal nerve fiber layer. Irregular vitreo-retinal interface with traction by partial PVD together with moderate hyperreflective dots in the vitreous.

Fluorescein angiography showed hyperfluorescence corresponding to the peripheral whitening with perivascular leakage in the left eye and optic disc leakage in both eyes (Figure 2A,B,C). B-scan of the superonasal quadrant did not reveal a corresponding elevation or abnormality (Figure 3A,B). OCT of the lesion revealed retinal infiltration with hyperreflective dots (Figure 4).

Anterior chamber paracentesis was performed at the slit lamp and sent for herpes simplex virus (HSV) and varicella zoster virus (VZV) PCR and gram stain. Other lab tests were ordered including complete blood count, complete metabolic panel, erythrocyte sedimentation rate, C-reactive protein, syphilis serology, HSV and VZV titer, human immunodeficiency virus (HIV) antibodies, chest X-ray, and MRI of the brain and orbit with and without contrast. The patient was not treated with steroids while waiting for the laboratory results to return.

Acute-phase reactant was elevated, HIV antibodies were negative, and rapid plasma regain (RPR) and fluorescent treponemal antibody absorption (FTA-Abs) were both reactive. After the diagnosis was confirmed to be syphilitic uveitis, the patient was admitted for high-dose intravenous penicillin G 24 million units per day. Lumbar puncture was recommended to evaluate for CSF antibodies, but the patient declined this invasive test. After the complete 2-week course of parenteral therapy, her ocular findings resolved dramatically.

### Discussion

Uveitis is the most common ocular manifestation of syphilis and is bilateral in more than 50% of cases; however, syphilitic uveitis is considered a rare cause of uveitis, accounting for 1.6% to 4.5% of cases [8].

The most common presentation of syphilitic uveitis varies between several reports [5,9,10]. According to a review article of 143 patients with syphilitic uveitis, the most common presentation is posterior uveitis followed by panuveitis [11]. Panuveitis, as seen in our case, most commonly occurs during the second stage of syphilis. Although the presentation may vary greatly between affected patients, there are certain features considered to be characteristic of syphilitic uveitis. As in our case, ground glass retinal opacification associated with retinal vasculitis is considered to be characteristic for syphilitic uveitis. Another distinctive feature described is acute syphilitic posterior placoid chorioretinitis (ASPPC) [12].

Standard testing used to screen for syphilis include non-treponemal tests of Venereal Disease Research Laboratory (VDRL) and RPR labs; however, these tests are nonspecific and may yield false positive results due to cross-reactivity. The gold standard tests used to confirm infection include FTA-Abs and dark field microscopy of the tissue [3]. Additionally, syphilis increases the risk of HIV transmission by two to five times, and co-infection is common; therefore, every patient diagnosed with syphilis should also be tested for HIV [11].

Since the optic nerve and retina are considered to be extensions of the CNS, ocular syphilis is regarded as a variant of neurosyphilis; thus, every patient with syphilitic uveitis should undergo lumbar puncture and CSF analysis for the detection of neurological involvement [13]. However, some authors argue that this is only necessary with neurological symptoms or higher RPR titre values [14]. Cerebrospinal fluid findings indicative of tertiary syphilis include greater than five white blood cells per microliter, elevated CSF protein levels, and treponemal or non-treponemal antibodies [15].

According to the CDC, Nebraska is ranked 48 among 50 states reporting at least one case of primary and secondary syphilis with 0.4 cases per 100,000 populations compared to the U.S. rate of 5.0. The rate among males was 0.8 cases per 100, 000 population compared to the U.S. male rate of 9.3. The rate among females was 0.1 compared to the U.S. female rate of 0.9 which show how rare syphilis is in this particular area.

The CDC recommends high-dose IV penicillin G 18 to 24 million units per day for 10 to 14 days. For HIV-positive patients, they also recommend an additional treatment of intramuscular benzathine penicillin at a dose of 2.4 million units weekly for 3 weeks. If severe penicillin allergic, one can consider ceftriaxone, oral doxycycline, or azithromycin. The Jarisch-Herxheimer reaction (JHR) can occur in up to a third of neurosyphilis patients following penicillin therapy [16]. The reaction usually includes fever, sweating, and temporary worsening of symptoms of disease. Some authors suggest the use of steroids prior to antibiotics in cases of severe neurosyphilis to prevent JHR [16].

Despite the rarity of the disease in certain areas, syphilis serology should be routinely done in every case of uveitis that requires investigation. Intravenous penicillin G is a highly effective treatment resulting in a dramatic improvement [17]; thus, early diagnosis and prompt treatment of syphilitic uveitis prevents potential irreversible complications.

## Consent

Written informed consent was obtained from the patient.

## Abbreviations

CDC: Centers for Disease Control and Prevention
OCT: optical coherence tomography
PCR: polymerase chain reaction
